# Vaccine Inoculation

**Published:** 1806-06

**Authors:** Joseph Brandreth

**Affiliations:** Chairman


### VACCINE INOCULATION.

AT a numerous meeting of the Medical Gentlemen of Liverpool, convened in the Board Room of the Infirmary, on Tuesday the 29th of April, 1806, for the purpose of taking into consideration certain Resolutions on the subject of Vaccine Inoculation, which had been previously drawn up by a Committee, consisting of the following gentlemen, viz. Dr. Rutter, Dr. Lyon, Dr. Bostock, Dr. Briggs, Mr. Park, and Mr. Dawson.

Dr. BRANDRETH in the Chair.

It was Resolved, unanimously,

1st. That the subjoined declaratory Resolutions be adopted.

2dly, That the said Resolutions be presented to those professional gentlemen in town, who were not present at the meeting, for their sanction and signatures; that when these signatures are obtained, the proceedings of this meeting, together with the Resolutions, be inserted once in each of the Liverpool newspapers; and that 2000 copies of the proceedings and Resolutions be printed for general circulation.

3dly, That the thanks of this meeting be given to the Committee above named, for their trouble in drawing up the said Resolutions.

4thly, That the thanks of this meeting be given to Dr. Brandreth, for his cool and patient attention and conduct in the Chair, during a long discussion of this important subject.

JOSEPH BRANDRETH, Chairman.

---

### RESOLUTIONS.

